# Supplementary material for: A Fundamental Study on a Porous Carbon Nanotubes Macroelectrode in Weakly Supported Electrolyte: A Novel Criterion for Distinguishing Diffusion Domains
Source: Int J Mol Sci. 2025 Aug 26;26(17):8262. doi: 10.3390/ijms26178262 (PMC12427835; doi:10.3390/ijms26178262)
Supplement: Supplementary file 1 [file ijms-26-08262-s001.zip › ijms-3807307-supplementary.pdf]

International Journal of
Molecular Sciences

MDPI

Supplementary Materials

A Fundamental Study on a Porous Carbon Nanotubes Macroelectrode in Weakly Supported Electrolyte: A Novel Criterion for Distinguishing Diffusion Domains

Josipa Dugeč, Ivana Škugor Rončević, Nives Vladislavić and Marijo Buzuk *

Department of General and Inorganic Chemistry, Faculty of Chemistry and Technology, University of Split, 21000 Split, Croatia; josipa.dugec@ktf-split.hr (J.D.); skugor@ktf-split.hr (I.Š.R.); nives@ktf-split.hr (N.V.)
* Correspondence: buzuk@ktf-split.hr; Tel.: +385-21-329-474

 https://doi.org/10.3390/ijms26178262

Supplementary Figures:

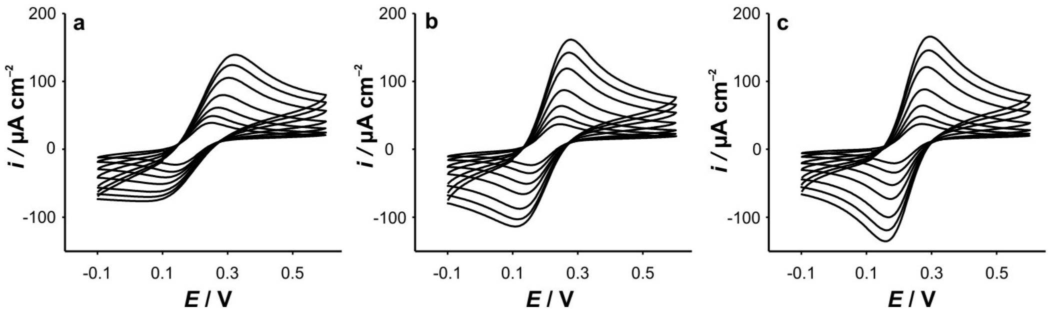

Figure S1. Cyclic voltammograms (3rd scan) for GCE at 0.5 mM Fe(II) in different KCl concentrations: (a) 0.001 M, (b) 0.01 M, and (c) 0.1 M KCl. Voltammograms were recorded at scan rates of 12.5, 25, 50, 100, 200, 300, and 400 mV s^{-1}.

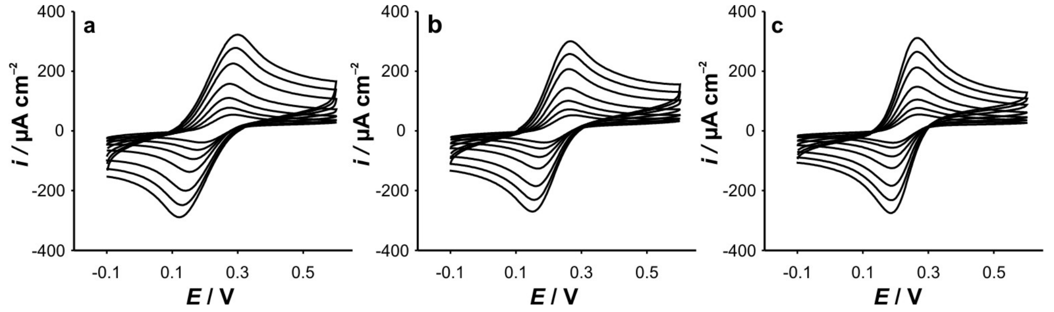

Figure S2. Cyclic voltammograms (3rd scan) for SWCNTs electrode at 0.5 mM Fe(II) in different KCl concentrations: (a) 0.001 M, (b) 0.01 M, and (c) 0.1 M KCl. Voltammograms were recorded at scan rates of 12.5, 25, 50, 100, 200, 300, and 400 mV s^{-1}.

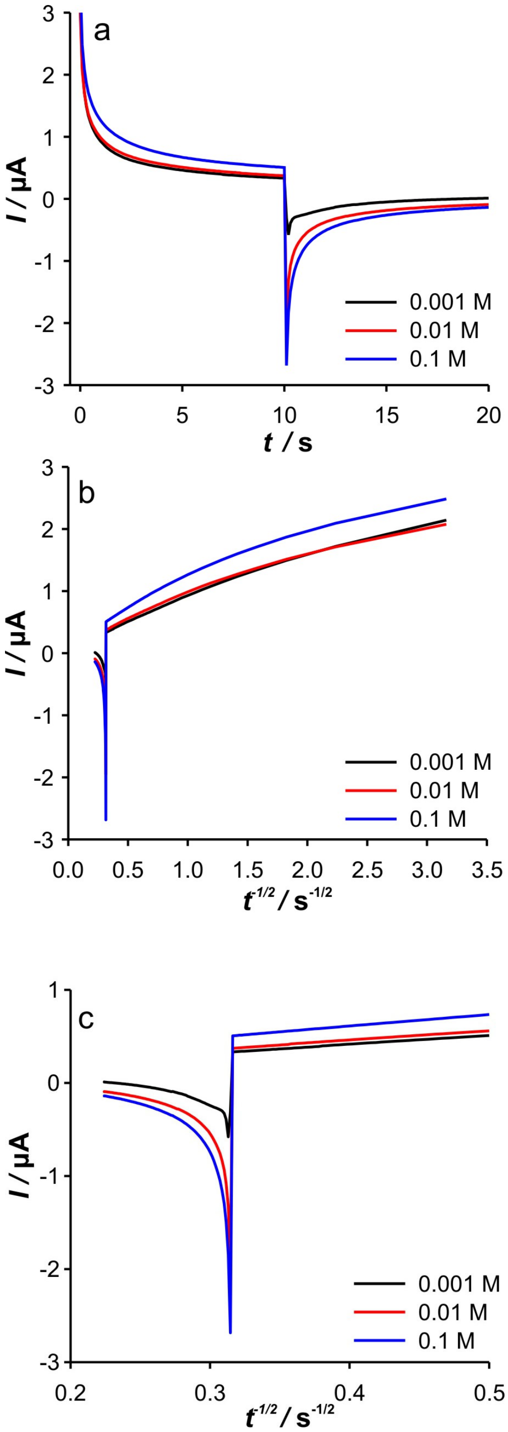

Figure S3. (a) DPSCAs obtained with SWCNTs electrode at 0.1 mM of Fe(II) in different KCl concentrations. Dependence of the current on $t^{-1/2}$: (b) the both branch of DPSCAs; (c) the cathodic branch of DPSCAs. All dependences are based on faradic currents and were derived from DPSCAs presented in (a).

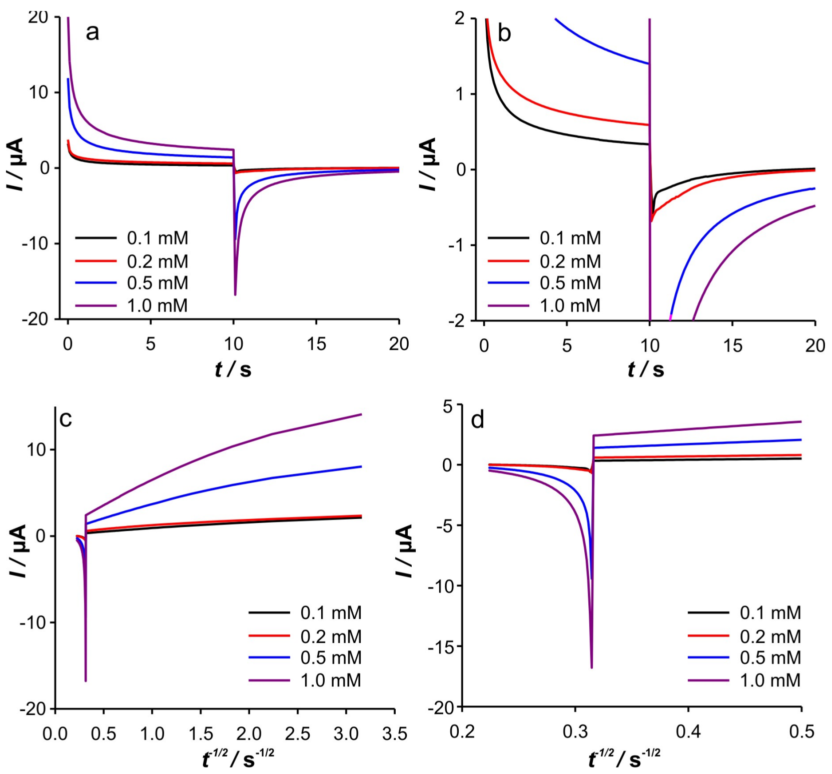

Figure S4. (a) DPSCAs obtained with SWCNTs electrode in 0.001 M KCl, at different concentrations of Fe(II); (b) Enlarged DPSCAs from (a). Dependence of the current on $t^{-1/2}$: (c) the both branch of DPSCAs; (d) the cathodic branch of DPSCAs. All dependences are based on faradic currents and were derived from DPSCAs presented in (a).

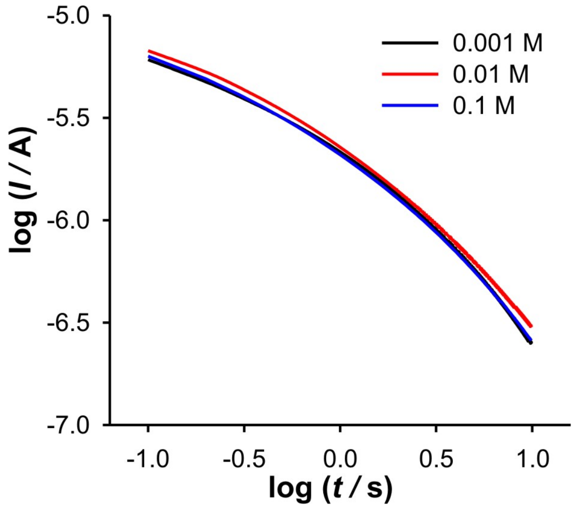

Figure S5. log(*i*) vs. log(*t*) dependences for SWCNTs electrode, calculated from the DPSCAs recorded at 0.5 mM of Fe(II) in different concentrations of KCl for the cathodic branch of DPSCAs

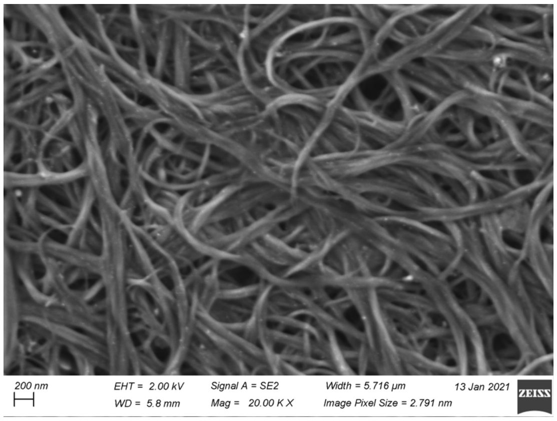

Figure S6. SEM images of the drop-casted SWCNTs layer on a glassy carbon electrode (GCE).
